# Hereditary Angioedema Attacks Resolve Faster and Are Shorter after Early Icatibant Treatment

**DOI:** 10.1371/journal.pone.0053773

**Published:** 2013-02-04

**Authors:** Marcus Maurer, Werner Aberer, Laurence Bouillet, Teresa Caballero, Vincent Fabien, Gisèle Kanny, Allen Kaplan, Hilary Longhurst, Andrea Zanichelli, on behalf of I. O. S. Investigators

**Affiliations:** 1 Department of Dermatology and Allergy, Allergie-Centrum-Charité, Charité - Universitätsmedizin Berlin, Berlin, Germany; 2 Department of Dermatology and Venerology, Medical University of Graz, Graz, Austria; 3 National Reference Centre for Angioedema, Internal Medicine Department, Grenoble University Hospital, Grenoble, France; 4 Hospital La Paz Health Research Institute (IdiPaz), Biomedical Research Network on Rare Diseases (CIBERER, U754), Madrid, Spain; 5 Shire Human Genetic Therapies, Eysins, Switzerland; 6 EA3999 ‘Allergic Disease: Diagnosis and Therapeutic’, University of Lorraine, Faculty of Medicine, Vandoeuvre-les-Nancy, France; 7 Medical University of South Carolina, Charleston, South Carolina, United States of America; 8 Department of Immunology, Bart’s and The London Hospital, London, United Kingdom; 9 Department of Clinical Sciences, Luigi Sacco University of Milan - Ospedale Luigi Sacco Milano, Milan, Italy; University of Leicester, United Kingdom

## Abstract

**Background:**

Attacks of hereditary angioedema (HAE) are unpredictable and, if affecting the upper airway, can be lethal. Icatibant is used for physician- or patient self-administered symptomatic treatment of HAE attacks in adults. Its mode of action includes disruption of the bradykinin pathway via blockade of the bradykinin B_2_ receptor. Early treatment is believed to shorten attack duration and prevent severe outcomes; however, evidence to support these benefits is lacking.

**Objective:**

To examine the impact of timing of icatibant administration on the duration and resolution of HAE type I and II attacks.

**Methods:**

The Icatibant Outcome Survey is an international, prospective, observational study for patients treated with icatibant. Data on timings and outcomes of icatibant treatment for HAE attacks were collected between July 2009–February 2012. A mixed-model of repeated measures was performed for 426 attacks in 136 HAE type I and II patients.

**Results:**

Attack duration was significantly shorter in patients treated <1 hour of attack onset compared with those treated ≥1 hour (6.1 hours versus 16.8 hours [*p*<0.001]). Similar significant effects were observed for <2 hours versus ≥2 hours (7.2 hours versus 20.2 hours [*p*<0.001]) and <5 hours versus ≥5 hours (8.0 hours versus 23.5 hours [*p*<0.001]). Treatment within 1 hour of attack onset also significantly reduced time to attack resolution (5.8 hours versus 8.8 hours [*p*<0.05]). Self-administrators were more likely to treat early and experience shorter attacks than those treated by a healthcare professional.

**Conclusion:**

Early blockade of the bradykinin B_2_ receptor with icatibant, particularly within the first hour of attack onset, significantly reduced attack duration and time to attack resolution.

## Introduction

Attacks of hereditary angioedema (HAE), a rare autosomal dominant disease, are caused by mutations in the C1-inhibitor (C1-INH) gene resulting in reduced plasmatic levels (HAE type I) or a dysfunctional protein (HAE type II). In the past few years a number of new treatment options have become available in the EU [1]. The identification of bradykinin as the key mediator of symptoms of HAE, and demonstration of its production at the sites of swelling [2], [3] led to the clinical development of icatibant, a bradykinin B_2_ receptor antagonist, as a treatment option for HAE type I and II [4], [5]. Icatibant is now licensed in 38 countries worldwide for subcutaneous healthcare professional (HCP)- or self-administration, to treat attacks of HAE type I and II in adults. Preliminary evidence in the literature suggests that early treatment of HAE attacks can shorten the duration of attacks, most likely because it reduces attack-to-treatment time [6], [7]. Additionally, early treatment has been demonstrated to reduce the time to relief of symptoms [8], [9]. The precise effect of early treatment on the course and duration of the attack in HAE patients, however, is unknown and remains to be investigated in detail. This analysis explores the impact of the timing of icatibant administration on the duration and resolution of HAE type I and II attacks.

Analyses and comparisons of the early and late treatment of HAE attacks in the past have been difficult for many reasons, including the fact that most HAE patients with attacks who required treatment would seek the help of physicians [6], [7]. The majority of HAE patients using icatibant are self-treaters and, because of this, early (within 2 hours of attack onset) and very early (within 1 hour of attack onset) treatment of HAE attacks has become more common. It is therefore now possible to compare sufficiently large numbers of HAE attacks treated at early and later timepoints.

The IOS (Icatibant Outcome Survey) database, which documents the routine clinical outcome of HAE patients treated with icatibant, is ideally suited to assess early attack treatment for differences in attack outcomes. Here, we have used the IOS data from more than 400 attacks in more than 100 patients to determine if early treatment by blockage of the bradykinin B_2_ receptor results in faster resolution and shorter duration of HAE attacks.

## Materials and Methods

### Participants

At the time of analysis, data from 310 patients with HAE type I or II were documented in the IOS database (baseline patient characteristics are shown in Table 1), with complete datasets available for 426 attacks from 136 patients (used in the analyses presented here). Of these 426 attacks, 233 attacks were self-treated and 174 attacks were treated by a HCP (data on who administered icatibant are missing for 19 attacks).

**Table 1:** Patient demographics.

HAE Diagnosis			
	Type I	Type II	All
**Gender**			
Male	115	6	121
Female	175	14	189
**Ethnicity**			
Asian	2	–	2
Black	1	2	3
Caucasian	272	18	290
Other†	9	–	9
Missing	6	–	6
**Gender**			
	**Male**	**Female**	**All**
**Country of residence**			
Austria	4	5	9
Germany	20	29	49
Denmark	13	6	19
Spain	23	39	62
France	35	68	103
Israel	1	–	1
Italy	16	27	43
Sweden	-	2	2
United Kingdom	9	13	22

† Other includes the total number of patients in the following categories: Other, n = 5; Caucasian and oriental, n = 1; Hispanic, n = 1; Jewish, n = 1; Mixed race, n = 1; South American, n = 1.

At the baseline visit, 85 patients (82 [96.5%] type I and 3 [3.5%] type II) were using long-term prophylaxis treatment. Of these, 40 (47.1%) patients were male and 45 (52.9%) were female. Concomitant and/or rescue medications, other than icatibant, taken by the patients for their attacks are shown in Table 2. No psychometric data were collected. The attack history of the 136 patients (mean, SD and median) over the previous 12 months collected at the baseline visit are summarized in Table 3.

**Table 2:** Concomitant and/or rescue medication taken by the patient for the attack.

Medication	Number of patients†	Total number of attacks
	(n = 136)	(n = 426)
C1-INH concentrate‡	18	68
Analgesics	9	14
Antifibrinolytics	7	12
Corticosteroids	5	5
Anti-emetics	2	7
Antihistamines	2	2
Attenuated androgens	2	2
Fluids	2	2
Spasmolytics	2	2
Other	2	7
Antacids	1	1
Anxiolytics/sedatives	1	2
Epinephrine	1	1
Fresh frozen plasma	0	0

† Patients could be counted in more than one category.

‡ Two patients were receiving long-term prophylaxis. For the remaining 16 patients, it is unknown whether they were using C1-INH concentrate as long-term prophylaxis or as rescue medication.

**Table 3:** Abdominal and cutaneous attack history from the previous 12 months for HAE type I and II patients.

		n	Mean (SD)	Median
**Abdominal**	Duration of untreated attacks (hours)	93	55.4 (28.2)	48.0
	Duration of treated attacks (hours)	140	27.6 (31.2)	17.0
	Number of attacks in previous 12 months	271	7.7 (14.3)	2.0
**Cutaneous**	Duration of untreated attacks (hours)	133	59.2 (31.3)	48.0
	Duration of treated attacks (hours)	125	32.9 (28.8)	24.0
	Number of attacks in previous 12 months	274	9.2 (15.8)	4.0

### Study Design and Setting

IOS is an on-going international, multicenter, prospective, observational study, currently being conducted at 38 centers in nine countries (Austria, Denmark, France, Germany, Israel, Italy, Spain, Sweden and the United Kingdom). This analysis is based on data collected between July 2009 and February 2012. As a non-interventional registry for patients treated with icatibant, as well as providing safety data, IOS is capturing prospective data on clinical outcomes for patients treated with icatibant.

IOS is conducted in accordance with the Declaration of Helsinki, and the International Conference on Harmonization Good Clinical Practice Guidelines. After approval from local Ethics Committees and/or Health Authorities (where applicable) had been obtained by the center, all patients provided written informed consent (Table S1). Icatibant is not licensed for use in HAE patients younger than 18 years. In the United Kingdom, for patients under 18, consent from the parents or legal representative was provided (Table S1).

### Study Visits

Following enrollment, data were collected from the patient during routine visits at 6-month intervals; including a physical exam, details relating to icatibant treatment (i.e. treatment of attacks over the previous months), concomitant medications and adverse events. Data collection was via physician-completed electronic survey forms delivered over a secure server.

Patient demographics and characteristics (including documentation of HAE or other diagnosis, general information on HAE attacks, previous treatments of HAE attacks), significant medical history, background data and medical history were collected at time of patient enrollment, and recorded by the attending physician.

At patient enrollment, each HAE attack treated with icatibant was described. The date and time of HAE attack, time of icatibant treatment(s) and time to complete resolution of the attacks were recorded. A description of the affected sites (skin, abdomen, larynx etc.), the severity of the attacks and type of administration (HCP- or self-administration) were also collated.

### Data Analyses

**Figure 1:**
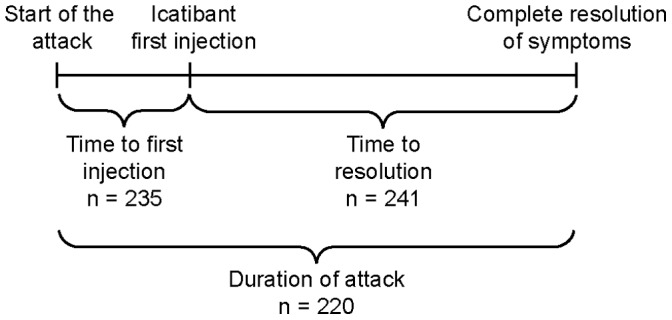
Available time-to-event data for patients with HAE types I and II.^†^ ^ †^For each attack experienced by patients in the IOS database, data were collected for time to first injection (time between start of the attack and icatibant first injection [n = 235 attacks]), time to resolution (time between icatibant first injection and complete resolution of symptoms [n = 241 attacks]) and duration of attack (time between start of the attack and complete resolution of symptoms [n = 220 attacks]).

For a patient’s attack, data were collected for time to first injection (defined as the time between start of attack and time of icatibant injection), time to resolution (defined as the time between first injection of icatibant and time to complete resolution of symptoms) and duration of attack (defined as the time between the start of attack to time to complete resolution of symptoms) (Figure 1). The time to events for patients with HAE type I and II were calculated only when the date and time of the events were available.

In order to streamline the reporting of the results, the median values are reported in the text, whilst mean and *p*-values are reported in the figures.

### Statistical Analyses

To test the hypothesis that early administration of icatibant is associated with reduced attack duration, the mean, median (and standard error of the mean) total duration of the attack was compared for attacks treated <1 hour versus ≥1 hour, <2 hours versus ≥2 hours, and <5 hours versus ≥5 hours of attack onset. Additionally, mean and median times to resolution following treatment were also assessed. The analyses include patients who self-administered icatibant and those who were treated by a HCP.

To compare the time to first treatment, the time to resolution and the duration of attack, a mixed-model analysis of repeated measures was used (Proc Mixed – SAS Institute, Cary, NC). A mixed-model is used to test for differences between two or more independent groups, whilst subjecting participants to repeated measures. The advantage of using a mixed model is that all the data available can be used. If there are data missing, it has no effect on other data available from the same patient. The Chi-square test was used for the comparison of percentages and the level of statistical significance chosen was alpha = 0.05.

## Results

### Early Treatment Resulted in Shorter Attack Duration than Late Treatment

**Figure 2:**
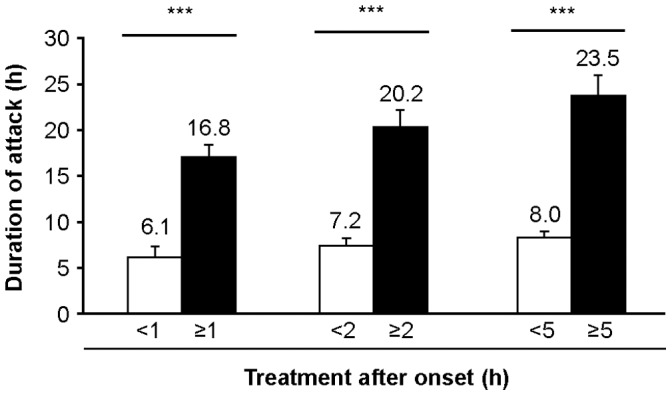
Early treatment reduced mean attack duration. Figure demonstrates the mean duration (time from the start of the attack to the complete resolution of symptoms) of attacks treated before and after each timepoint. n = 207 attacks (<1 hour, n = 80; ≥1 hour, n = 127; <2 hours, n = 120; ≥2 hours, n = 87; <5 hours, n = 145; ≥5 hours, n = 62); all attacks are included at each timepoint; ***p<0.001.

Attack duration was significantly shorter in attacks treated <1 hour of onset (median 2.0 hours) when compared with attacks treated ≥1 hour (median 14.0 hours; *p*<0.001) (Figure 2). Attacks were also shorter when treated <2 hours of onset (median 2.5 hours) as compared with ≥2 hours after onset (median 16.0 hours; *p*<0.001), as were attacks treated <5 hours of onset (median 3.5 hours) compared with attacks treated ≥5 hours (median 17.8 hours; *p*<0.001) (Figure 2). Comparison of the mean duration of action demonstrate that at 1 hour, 2 hours and 5 hours, the attacks were 2.74-, 2.82- and 2.94-fold longer, respectively, than those treated earlier (Figure 2). The significant reduction in attack duration associated with earlier treatment was observed for both self- and HCP-treated attacks (data not shown).

Both abdominal and cutaneous attacks were found to be of shorter total duration when treated early. Additionally, early treatment resulted in significant reductions in attack duration regardless of attack severity (Tables 4 and 5).

**Table 4:** Early treatment with icatibant and mean attack duration by attack location.

	Time from attack onsetto treatment	Number of attacks	Mean attack duration (SD)	Median	*p*-value
**Abdominal**	<1 hour	36	6.5 (9.4)	2.0	
	≥1 hour	69	14.6 (11.5)	12.0	<0.001
	<2 hours	63	8.3 (10.6)	2.1	
	≥2 hours	42	17.1 (10.8)	14.4	<0.001
	<5 hours	76	9.7 (11.5)	4.6	
	≥5 hours	29	17.4 (9.7)	15.0	0.002
**Cutaneous**	<1 hour	27	5.7 (12.2)	1.3	
	≥1 hour	43	20.3 (23.4)	15.3	0.004
	<2 hours	37	5.7 (10.7)	2.0	
	≥2 hours	33	24.7 (25.1)	21.0	<0.001
	<5 hours	47	6.0 (9.6)	3.0	
	≥5 hours	23	32.3 (26.6)	24.0	<0.001

**Table 5:** Early treatment with icatibant and mean attack duration by attack severity.

	Time from attack onsetto treatment	Number of attacks	Mean attack duration (SD)	Median	*p*-value
Mild-to-moderate	<1 hour	19	3.7 (4.6)	1.8	
	≥1 hour	24	14.8 (15.3)	11.0	0.004
	<2 hours	24	3.4 (4.1)	2.0	
	≥2 hours	19	18.1 (15.6)	12.2	<0.001
	<5 hours	30	4.2 (4.3)	2.3	
	≥5 hours	13	23.1 (16.6)	21.0	<0.001
Severe	<1 hour	61	6.9 (10.8)	2.0	
	≥1 hour	101	17.3 (16.9)	14.5	<0.001
	<2 hours	95	8.1 (10.8)	2.6	
	≥2 hours	67	20.7 (18.5)	16.0	<0.001
	<5 hours	114	9.0 (11.1)	4.5	
	≥5 hours	48	23.6 (19.9)	17.0	<0.001

### HAE Attacks Treated within 1 Hour of Attack Onset Resolved Faster than those Treated after 1 Hour

**Figure 3:**
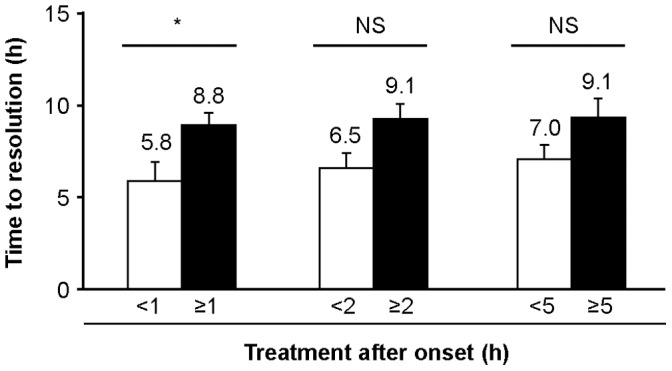
Very early treatment with icatibant reduced mean time to resolution. Figure demonstrates the mean time to resolution (time between first injection and complete resolution of symptoms) of attacks treated before and after each timepoint. n = 207 attacks (<1 hour, n = 80; ≥1 hour, n = 127; <2 hours, n = 120; ≥2 hours, n = 87; <5 hours, n = 145; ≥5 hours, n = 62); all attacks are included at each timepoint; *p = 0.033, NS, not significant.

Attacks treated within 1 hour of attack onset resolved significantly faster than those treated after 1 hour (median 1.7 hours versus median 6.0 hours; *p* = 0.033; Figure 3). Attacks treated after the first hour of onset, on average, took 3 more hours to resolve than attacks treated during the first hour, indicating an increase of >50%. Treatment within 2 or 5 hours of attack onset also resulted in faster, albeit not statistically significantly, resolution of attacks compared with treatment after 2 or 5 hours (Figure 3). Attacks treated after the second and fifth hour of attack onset, on average, took 2.5 (+40%) and 2.1 (+30%) more hours to resolve than attacks treated during the first 2 and 5 hours, respectively.

### Self-administration Resulted in Earlier Treatment and Shorter Attack Duration than HCP Treatment

**Figure 4:**
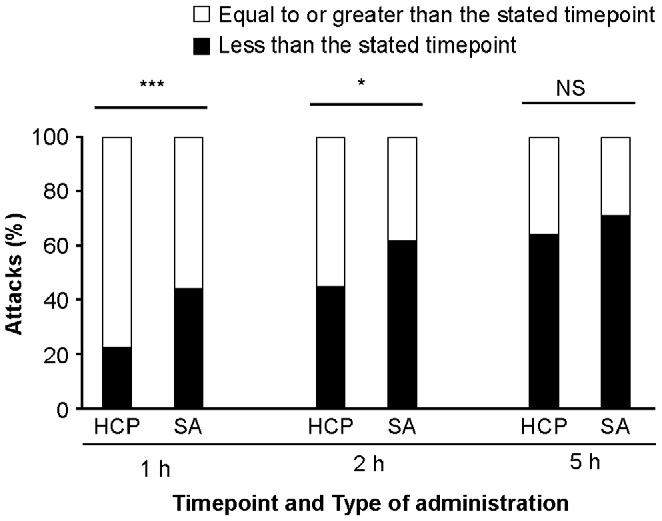
Self-administration of icatibant resulted in earlier treatment. Figure shows proportion of patients treated before and after each timepoint n = 72 for HCP-administered patients, n = 158 for self-administered patients. All patients are included at each timepoint; *** *p* = 0.001 for self-administration versus HCP-administration,*p = 0.016 for self-administration versus HCP-administration, NS, not significant; HCP, healthcare professional; SA, self-administration.

The proportion of attacks treated within 1 hour after attack onset was twice as high for self-administrators as for those who received HCP-administered icatibant (44% attacks versus 22% attacks, respectively; *p* = 0.001) (Figure 4). The majority of attacks in self-treating patients (>60%) were treated within the first 2 hours, compared with less than half of attacks in HCP-treated patients (44%; *p* = 0.016).

**Figure 5:**
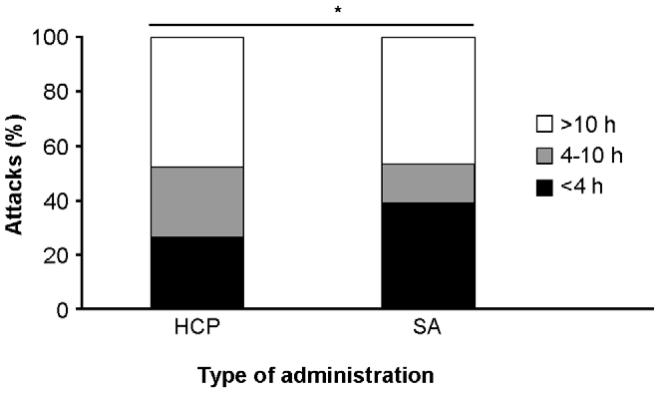
Self-administration of icatibant resulted in shorter duration of attack. Figure shows duration of attack by type of administration. n = 17, 17 and 32 for attacks lasting <4 hours, 4–10 hours and ≥10 hours for HCP-administered patients, respectively; n = 57, 20 and 69 for attacks lasting <4 hours, 4–10 hours and ≥10 hours for self-administered patients; *p = 0.047 for self-administration versus HCP-administration; HCP, healthcare professional; SA, self-administration.

Self-treated attacks were of shorter duration than HCP-treated attacks (*p*<0.05). For attacks lasting up to 4 hours, between 4 and 10 hours and greater than 10 hours, the proportion of attacks that were HCP-treated versus self-treated were 25.8% versus 39.0%, 25.8% versus 13.7% and 48.5 versus 47.3%, respectively (Figure 5).

## Discussion

Here, we demonstrate for the first time that early treatment of HAE attacks is more beneficial for patients. Historically, patients with HAE attacks have been advised to wait until the attack became moderate in severity before seeking treatment, and it is only recently that this advice has been revised. The Hereditary Angioedema International Working Group (HAWK) consensus recommends early on-demand treatment for all HAE attacks, regardless of location and ideally before visible or disabling symptoms develop [10]. Up until now the evidence for this recommendation, however, was weak.

Our findings demonstrate that the earlier patients with attacks of HAE type I and II receive icatibant (particularly within 1 hour of attack onset), the shorter the attack duration, irrespective of attack location (abdominal or cutaneous) or severity. Previous preliminary studies demonstrated that earlier treatment with C1-INH was associated with shorter attack duration [6]–[9]. During our investigation, when looking closely at the impact of early treatment on attack duration, it can be observed that both early and very early treatment reduce attack duration. We therefore hypothesized that attacks that are still in early stages of development may respond more favorably to treatment than attacks that have developed further. Treating attacks later and allowing attacks to develop for more than an hour resulted in reduced response to icatibant treatment and delayed resolution by more than 50% (or 3 hours).

The beneficial effect of early treatment was also observed when comparing self- and HCP-administration on attack duration when icatibant was administered within 1 hour of attack onset. There was no statistically significant difference in the time to resolution between self- and HCP-administered icatibant; however, there was a statistically significant difference favoring self-administration in the duration of attacks. Patients with HAE can experience delays in receiving the correct treatment because HCPs are unfamiliar with HAE and its treatment. Attacks can impact patients’ work and social lives and can be life-threatening in the case of laryngeal edema. Self-administration facilitates early treatment, which shortened the duration of attacks and can reduce the need to seek help from HCPs for patients experiencing non-laryngeal attacks, all of which may have a positive impact on the management of HAE. Patients experiencing laryngeal attacks, however, should still seek medical attention following self-treatment.

The efficacy of icatibant observed in the IOS database is comparable to that observed in three Phase III clinical trials, For Angioedema Subcutaneous Treatment −1, −2 and −3 (almost complete symptom relief between 8.0 and 10.0 hours following treatment) [5], [11], confirming the efficacy of icatibant in a real-world setting.

The reasons for early treatment with icatibant being more effective than administration after a few hours are not clear, particularly when one considers that attacks of swelling can continue to evolve during the first 12–24 hours. Icatibant acts to block the effect of bradykinin on B_2_ receptors which are present constitutively along vascular (venular) endothelial cells, and icatibant has no direct effect on factor XII activation, no effect on activated factor XII on prekallikrein, and no effect on kallikrein activity. Thus, one could propose that there is somehow an amplification of the production of bradykinin that is a result of the interaction of bradykinin with vascular endothelial cells and which is inhibited by prompt administration of icatibant. Two possibilities are suggested given what we currently know about the plasma bradykinin-forming cascade. When endothelial cells are activated, they release nitric oxide, prostaglandins, and tissue plasminogen activator (TPA) [12]. The TPA converts plasminogen to plasmin, and plasmin can act to activate factor XII [13]. Although there is clearly feedback activation of factor XII by the resulting kallikrein, plasmin itself can accomplish this. Plasmin not only produces factor XIIa at 80 Kd but also further degrades the factor XIIa to factor XIIf at 28.5 Kd [14]. This effect may be particularly evident when there is very significant overproduction of bradykinin, for example during attacks of angioedema in patients with HAE. Thus, although conversion of factor XIIa to factor XIIf is readily demonstrable *in vitro* using purified enzymes, identification in factor XIIf in plasma has only been demonstrated when C1-INH is absent or dysfunctional [15]. A second possibility is that the activation of the plasma bradykinin-forming cascade on endothelial cells occurs in the absence of factor XII, as a result of heat shock protein (HSP-90) [16], thus catalyzing prekallikrein conversion to kallikrein. The kallikrein thus formed can cleave high molecular weight kininogen to produce bradykinin but, more importantly, can activate factor XII (along with plasmin), thereby maximizing the quantity of factor XII recruited into the process [17]. Factor XIIf lacks a surface (and cellular) binding site and can cause bradykinin formation at sites distant from the initial localized site of swelling. This facilitates the spread of angioedema to other distal areas. The theory of whether endothelial cell TPA and HSP-90 are released as a result of endothelial cell stimulation by bradykinin is an area of current investigation [18].

In conclusion, this analysis demonstrates that early treatment of HAE type I and II attacks with icatibant can significantly reduce the duration and time to resolution of HAE attacks. Our data provide evidence in support of recommendations to treat early and promote self-treatment. Our findings also call for in-depth investigations into how the early blockage of bradykinin B_2_ receptors improves the course of HAE attacks. This will help to improve treatment recommendations for HAE patients.

## Supporting Information

Table S1 Ethics Committee approvals.
(DOCX)
