# Supplementary material for: Hereditary Angioedema Attacks Resolve Faster and Are Shorter after Early Icatibant Treatment
Source: PLoS One. 2013 Feb 4;8(2):e53773. doi: 10.1371/journal.pone.0053773 (PMC3563637; doi:10.1371/journal.pone.0053773)
Supplement: Table S1 — Ethics Committee approvals. (DOCX) [file pone.0053773.s001.docx]

**Supplementary Table S1.**

Ethics Committee approvals

| **Country** | **Was Ethics Committees approval required?  If yes, by whom?** | **Were other approvals required?  If yes, by whom?** | **Was written informed consent for patients under 18 years required (Yes/No)?** |
| --- | --- | --- | --- |
| **Austria** | Yes; Ethik Kommission, Graz | No | Yes; however, no patients under 18 years were enrolled in Austria |
| **Denmark** | No | Yes; approval by the Data Protection Agency | Yes; however, no patients under 18 years were enrolled in Denmark. |
| **France** | No | Yes, approval by *Comité consultatif sur le traitement de l’information en matière de recherche dans le domaine de la santé* (CCTIRS) and *Commission nationale de l’informatique et deslibertés* (CNIL). | No specific informed consent required, however, for the one patient under the age of 18 years who was enrolled, informed consent was obtained from the parents. |
| **Germany** | Yes; Ethik Kommission, Berlin; Ethik Kommission, Mainz; Ethik Kommission, Essen; Ethik Kommission, Dresden; Ethik Kommission, Münschen | No | Yes; however, no patients under 18 years were enrolled in Germany. |
| **Israel** | Yes; Bnai-Zion Medical Center Ethics Committee, Haifa | No | Yes; however, no patients under 18 years were enrolled in Israel. |
| **Italy** | Yes; Comitato Etico Locale per la Sperimentazione, Milano; Comitato Etico Indipendente Locale, Bari; Comitato Etico per le Attività Biomediche, Napoli; Comitato Etico, Palermo; Comitato Etico per la Sperimentazione, Padova | No | Yes; however, no patients under 18 years were enrolled in Italy. |
| **Spain** | Yes; Comité Ético de Investigación Clínica Fundación Para La Investigación Biomédica, Hospital Gregorio Marañón, Madrid; Comité Ético de Investigación Clínica, Hospital Universitario La Paz, Madrid; Comité Ético de Investigación Clínica, Hospital Universitario Vall d’Hebron, Barcelona; Instituto de Investigación Sanitaria, Hospital Universitario La Fe, Valencia | Yes*;* approval by *Agencia Española de Medicamentos y Productos Sanitarios* (AEMPS). | Yes; however, no patients under 18 years were enrolled in Spain. |
| **Sweden** | Yes; Regionala Etikprövningsnämnden, Linköping | No | Yes; one patient aged 17 years was enrolled. The patient and their parent signed an informed consent form. |
| **United Kingdom** | Yes; Central Manchester Research Ethics Committee, Manchester | No | Yes; one patient aged 17 years was enrolled. The patient and their parent signed an informed consent form. |
